# Establishment of a new human pleomorphic malignant fibrous histiocytoma cell line, FU-MFH-2: molecular cytogenetic characterization by multicolor fluorescence in situ hybridization and comparative genomic hybridization

**DOI:** 10.1186/1756-9966-29-153

**Published:** 2010-11-24

**Authors:** Jun Nishio, Hiroshi Iwasaki, Kazuki Nabeshima, Masako Ishiguro, Teruto Isayama, Masatoshi Naito

**Affiliations:** 1 Department of Orthopaedic Surgery, Faculty of Medicine, Fukuoka University, 7-45-1 Nanakuma, Jonan-ku, Fukuoka 814-0180, Japan; 2 Department of Pathology, Faculty of Medicine, Fukuoka University, 7-45-1 Nanakuma, Jonan-ku, Fukuoka 814-0180, Japan; 3 Department of Orthopaedic Surgery, Yanagawa Rehabilitation Hospital, 113-2 Kamimiyanaga-Machi, Yanagawa City, Fukuoka 832-0058, Japan

## Abstract

**Background:**

Pleomorphic malignant fibrous histiocytoma (MFH) is one of the most frequent malignant soft tissue tumors in adults. Despite the considerable amount of research on MFH cell lines, their characterization at a molecular cytogenetic level has not been extensively analyzed.

**Methods and results:**

We established a new permanent human cell line, FU-MFH-2, from a metastatic pleomorphic MFH of a 72-year-old Japanese man, and applied multicolor fluorescence in situ hybridization (M-FISH), Urovysion™ FISH, and comparative genomic hybridization (CGH) for the characterization of chromosomal aberrations. FU-MFH-2 cells were spindle or polygonal in shape with oval nuclei, and were successfully maintained *in vitro *for over 80 passages. The histological features of heterotransplanted tumors in severe combined immunodeficiency mice were essentially the same as those of the original tumor. Cytogenetic and M-FISH analyses displayed a hypotriploid karyotype with numerous structural aberrations. Urovysion™ FISH revealed a homozygous deletion of the *p16*^*INK4A *^locus on chromosome band 9p21. CGH analysis showed a high-level amplification of 9q31-q34, gains of 1p12-p34.3, 2p21, 2q11.2-q21, 3p, 4p, 6q22-qter, 8p11.2, 8q11.2-q21.1, 9q21-qter, 11q13, 12q24, 15q21-qter, 16p13, 17, 20, and X, and losses of 1q43-qter, 4q32-qter, 5q14-q23, 7q32-qter, 8p21-pter, 8q23, 9p21-pter, 10p11.2-p13, and 10q11.2-q22.

**Conclusion:**

The FU-MFH-2 cell line will be a particularly useful model for studying molecular pathogenesis of human pleomorphic MFH.

## Background

Pleomorphic malignant fibrous histiocytoma (MFH), also known as undifferentiated high grade pleomorphic sarcoma, is among the most common adult soft tissue sarcomas, but the precise histogenesis of this tumor is controversial [1]. Pleomorphic MFH frequently shows highly aggressive behavior, resistance to radiotherapy and chemotherapy, and fatal metastasis.

Well-characterized human sarcoma cell lines are valuable resources for developing new strategies against sarcoma cell growth and progression. Although a number of human cell lines derived from MFH have been reported [2-17], their characterization at the molecular cytogenetic level has been limited. Here, we describe the development of a new human cell line, designated as FU-MFH-2, derived from a metastatic pleomorphic MFH. In addition, we investigate genomic alterations in FU-MFH-2 by a combination of molecular cytogenetic techniques.

## Methods

### Source of tumor cells

**Figure 1:**
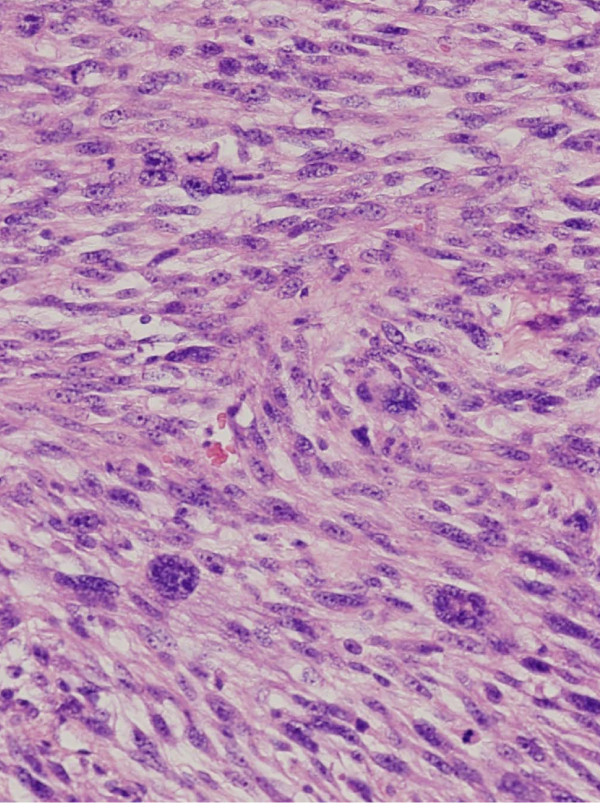
**Histologic appearance of the original tumor showing atypical spindle cells, polygonal cells, and bizarre giant cells, corresponding to pleomorphic MFH**.

The original tumor tissue specimen was surgically obtained from a metastatic pleomorphic MFH of the left thigh in a 72-year-old Japanese man (Figure 1). One year earlier, a left lower leg tumor was resected and a histological diagnosis of pleomorphic MFH was established. Immunohistochemically, the tumor cells were frequently positive for vimentin and focally for CD68 and lysozyme. The other antibodies tested were negative. The patient died of lung metastasis 2 years after the initial diagnosis.

### Establishment of cell line and determination of cell population doubling time

Fresh tumor tissue was minced with fine scissors and then digested with 200 IU/ml type II collagenase (Worthington Biochemical Corporation, Freehold, NJ, USA) in serum-free medium for 30 minutes at 37°C. After digestion, isolated cells were washed and seeded in a 25-cm^2 ^plastic flask (Falcon 3013, Becton Dickinson Japan, Tokyo, Japan) containing culture medium, and maintained in a humidified atmosphere of 5% CO_2 _in air at 37°C. The culture medium was composed of a 1:1 mixture of Dulbecco's Modified Eagle Medium (DMEM) and Ham's F-12 (GIBCO BRL, Grand Island, NY, USA) supplemented with 10-20% fetal calf serum (FCS; Cell Culture Laboratories, Cleveland, OH, USA) and kanamycin sulfate (100 μg/ml; Meiji Seika, Tokyo, Japan). The medium was replaced twice weekly. When semi-confluent layers were obtained, the cells were dispersed with phosphate buffered saline (PBS) containing 0.1% trypsin and 0.02% ethylenediamine tetraacetic acid (EDTA) solution and seeded in new flasks for passage. These procedures were serially performed until establishment of the FU-MFH-2 cell line.

To determine the doubling time, 1.0 × 10^5 ^FU-MFH-2 cells/cm^2 ^at passage 31 were seeded in each well of 24-well dishes (Corning Costar, Tokyo, Japan) with fresh medium containing 1 ml of DMEM/F-12 with 10% FCS. The culture dishes were harvested, and then the number of viable cells in each dish was counted by the dye exclusion test (0.1% trypan blue in PBS) every 24 hours for 7 days.

### Tumorigenicity in severe combined immunodeficiency (SCID) mice

To determine the tumorigenicity of the FU-MFH-2 cell line *in vivo*, 5 × 10^7 ^cells at passage 23 were washed, suspended in PBS, and injected subcutaneously into the back of two 5-week-old female athymic SCID mice (CB-17/Icr-scid; Jcl Clea Japan, Inc., Osaka, Japan). The mice were maintained in a pathogen-free environment and carefully observed after transplantation. The experimental protocol was approved by the Ethics Review Committee for Animal Experimentation of Fukuoka University Faculty of Medicine.

### Pathologic studies

The cells grown in culture flasks were observed by phase-contrast microscopy. FU-MFH-2 cells at passages 31 and 42 were examined. For routine light microscopy, the cells cultured in chamber slides (Lab-Tek, Miles Laboratories, Naperville, IL, USA) were fixed in methanol and stained with hematoxylin and eosin (H&E) and Giemsa. Paraffin sections from the original tumor and xenografts were stained with the same reagents. The primary antibodies and their dilutions used for immunocytochemistry are listed in Table 1. The cells grown in chamber slides were washed in PBS and fixed in cold acetone for 5 minutes. The cells were reacted with each of the primary antibodies for 1 hour at room temperature. The bound antibodies were then visualized using a labeled streptavidin biotin system and the alkaline phosphatase technique, as described previously [15]. Paraffin sections from the original tumor and xenografts were also examined using the same procedure.

**Table 1:** Antibodies used in the present study.

Antibody	Type	Source	Dilution
Vimentin	M	Dakopatts, Kyoto, Japan	1:50
EMA	M	Dakopatts	1:50
AE1/AE3	M	Dakopatts	1:50
CAM 5.2	M	Becton Dickinson, San Jose, CA, USA	1:50
Desmin	M	Dakopatts	1:50
α-SMA	M	Dakopatts	1:50
MSA (HHF35)	M	Enzo Diagnostics, Farmingdale, NY, USA	1:50
S-100 protein	P	Dakopatts	1:1000
NSE	M	Dakopatts	1:200
CD68 (KP-1)	M	Dakopatts	1:200
Lysozyme	P	Dakopatts	1:500
AAT	P	Dakopatts	1:1000
ACT	P	Dakopatts	1:1000
C-Kit	P	Immuno-Biological Laboratories, Fujioka, Japan	1:10

*Abbreviations: *EMA, epithelial membrane antigen; α-SMA, alpha-smooth muscle actin; MSA, muscle-specific actin; NSE, neuron-specific enolase; AAT, alpha-1-antitrypsin; ACT, alpha-1-antichymotrypsin; M, monoclonal (mouse); P, polyclonal (rabbit).

### Cytogenetic analysis

The FU-MFH-2 cells at passages 25 and 52 and the fresh original tumor cells were used for cytogenetic analysis. Metaphase cells were banded with Giemsa trypsin, and karyotypic descriptions were done according to the International System for Human Cytogenetic Nomenclature 2009 [18].

#### Multicolor fluorescence in situ hybridization (M-FISH) and Urovysion™ FISH

Multicolor and multitarget FISH studies were performed on unstained cytogenetic preparations utilizing a commercially available M-FISH probe kit (24XCyte, MetaSystems GmbH, Altlusheim, Germany) and the Urovysion™ FISH probe mixture (Abbott Molecular, Des Plaines, IL, USA) containing a specific probe for the locus 9p21 (Spectrum Gold) and three alpha-satellite centromere-specific probes for chromosomes 3 (Spectrum Red), 7 (Spectrum Green), and 17 (Spectrum Aqua), respectively. The cells and probes were codenatured at 72°C for 2 minutes and subsequently placed in a moist chamber for at least two nights at 37°C. Post-hybridization washing was performed as previously described with minor modifications [19,20]. The slides were air-dried in the dark and counterstained with 4',6-diamidino-2-phenylindole (DAPI II; Abbott Molecular). Image processing and 24-color karyotyping were performed with the SpectraVysion Imaging System (Abbott Molecular). Hybridization signals were assessed in a minimum of 10 metaphase cells.

### DNA extraction and Comparative genomic hybridization (CGH)

DNA was extracted from FU-MFH-2 cells at passage 25 and from the original tumor tissue according to a standard procedure using phenol and chloroform extraction followed by ethanol precipitation. The purity and molecular weight of DNA were estimated using ethidium bromide-stained agarose gels.

CGH was performed as described previously [21]. Briefly, DNA from the FU-MFH-2 cell line and original tumor was directly labeled with fluorescein-12-dUTP (Roche Diagnostics, Mannheim, Germany) by nick translation, with the use of a commercial kit (Abbott Molecular). As a normal reference DNA, we used the Spectrum Red directed-labeled male total human DNA (Abbott Molecular). Subsequently, equal amounts of normal and tumor labeled probes (400 ng) and 20 μg of Cot-1 DNA (GIBCO BRL) were coprecipitated with ethanol. The precipitated DNA was dissolved in 10 μl of hybridization buffer and denatured at 75°C for 8 minutes. Normal metaphase spreads (Abbott Molecular) were denatured for 3 minutes at 75°C and hybridized with the DNA mixture in a moist chamber for 3 days. Slides were washed according to the protocol supplied by the manufacturer. Chromosomes were counterstained with 4',6-diamino-2-phenylindole (DAPI; Sigma, St. Louis, MO, USA) and embedded in antifade solution (Vectashield, Vector Laboratories, Burlingame, CA, USA).

### Digital image analysis

The location of aberrant CGH signals was analyzed using an image analysis system (Isis, Carl Zeiss Vision, Oberkochen, Germany) based on an integrated high-sensitivity monochrome charge-coupled device camera and automated CGH analysis software (MetaSystems GmbH). Three-color images, green (fluorescein-12-dUTP) for the tumor DNA, red (Spectrum Red) for the reference DNA, and blue (DAPI) for the DNA counterstain, were acquired from at least 10 metaphases. Only metaphases of good quality with strong, uniform hybridization were included in the analysis. Based on the control experiments, 1.2 and 0.8 were used as cutoff levels for gains and losses, respectively. Gains exceeding the 1.5 threshold were termed high-level amplifications. The heterochromatic regions in chromosomes 1, 9, and 16, the p-arms of the acrocentric chromosomes, and Y chromosome were excluded from the analysis because of suppression of hybridization with Cot-1 DNA in these regions.

## Results

### Establishment of FU-MFH-2 cell line and doubling time

Four weeks after initial cultivation in primary culture, spindle-shaped, round, or polygonal tumor cells reached sub-confluence with some piled-up foci of cells. These cells were collected after a 5-minute digestion at 37°C with a 0.1% trypsin solution, and replated in two 25-cm^2 ^plastic flasks containing fresh medium. Once confluent they were serially subcultured at a dilution of 1:2. Approximately 2 months later, at passages 4 to 5, the cells began to grow rapidly, and thereafter could be serially subcultured at a dilution of 1:2 every week. This new cell line was designated FU-MFH-2, and has been maintained *in vitro *for more than 80 passages (a period of more than 12 months). The population-doubling time of FU-MFH-2 cells in logarithmic growth phase was approximately 56 hours.

### Tumor formation in vivo

Small elastic hard nodules became palpable in all SCID mice at approximately 4 weeks after inoculation of FU-MFH-2 cells. Two months later, the tumors had grown up to 2.2 cm in diameter. The cut surfaces of these tumors were solid and white with no secondary changes. The mice were sacrificed humanely, and no metastatic lesions were identified at autopsy.

### Morphologic characterization in vitro and in vivo

**Figure 2:**
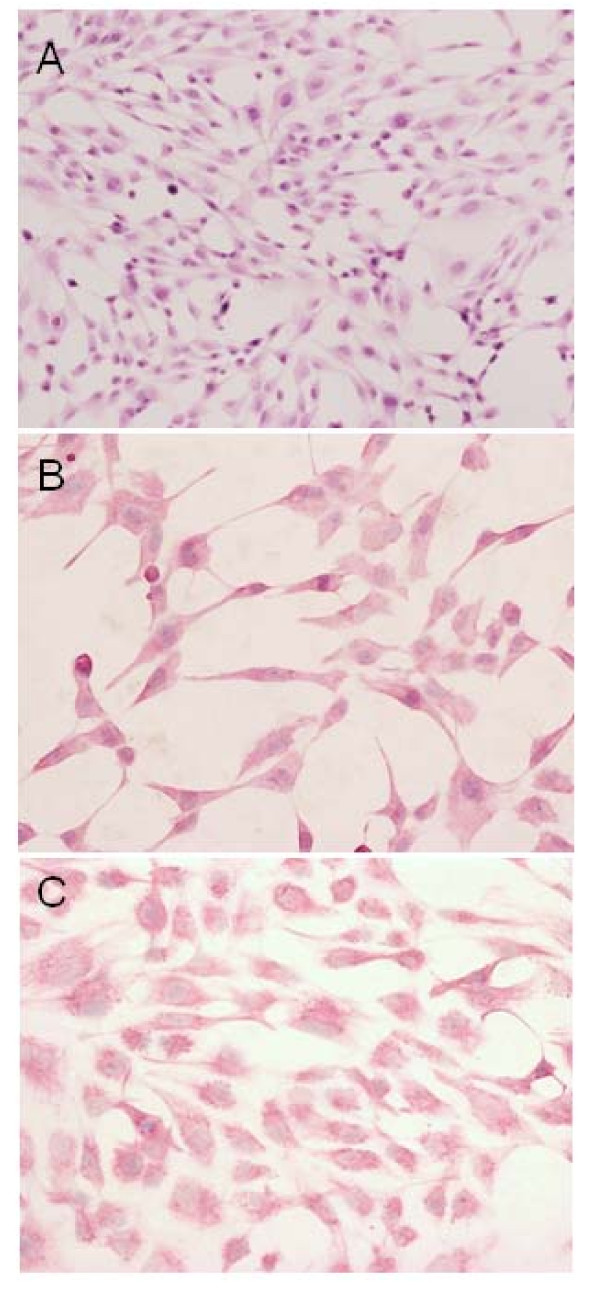
**Light microscopic findings of FU-MFH-2 cells *in vitro***. (A) FU-MFH-2 cells are spindle, round or polygonal in shape with oval nuclei and extension of slender cytoplasmic processes. Most FU-MFH-2 cells exhibit immunopositive reaction for vimentin (B) and CD68 (C).

**Figure 3:**
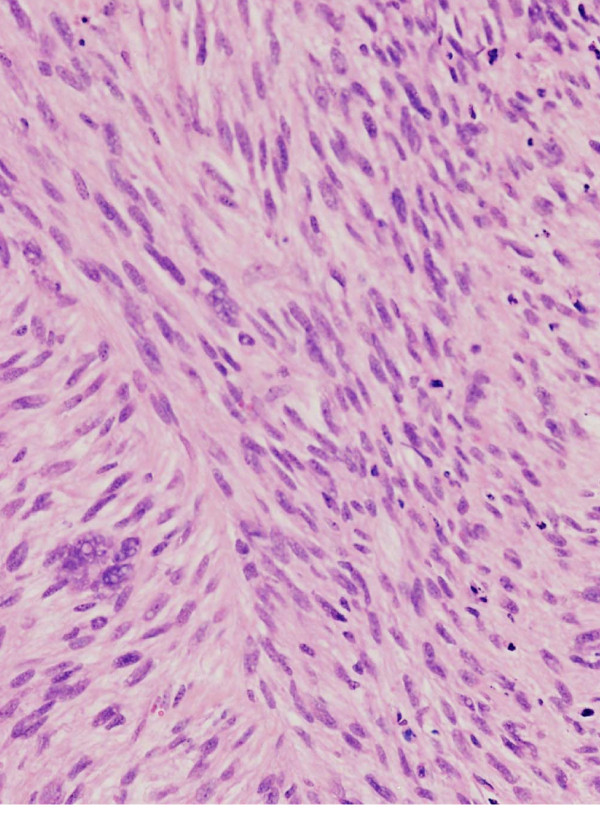
**Light microscopic finding of FU-MFH-2 cells *in vivo***. A representative portion of the tumor in a SCID mouse, essentially resembling the original tumor.

As assessed by light microscopy, FU-MFH-2 cells growing in chamber slides were spindle-shaped, round or polygonal with extended slender cytoplasmic processes. The cells proliferated loosely or in a storiform pattern accompanied by irregularly piled up foci. The nuclei were oval with distinct nucleoli (Figure 2A). As shown by immunocytochemistry (Table 2), these cells were positive for vimentin (Figure 2B) and CD68 (Figure 2C). The other antibodies tested *in vitro *were negative. On the other hand, the histological features of the heterotransplanted tumors were essentially similar to those of the original tumor. Namely, the tumors were composed of a mixture of atypical spindle cells, round cells, and bizarre giant cells arranged in a storiform pattern (Figure 3). Mitotic figures were frequently found. Immunohistochemically (Table 2), the tumor cells were positive for vimentin and focally for CD68, but were negative for the other antibodies tested *in vivo*.

**Table 2:** Reactivity of FU-MFH-2 cells, *in vitro *and *in vivo*, including the original tumor cells with various antibodies.

Antibody	FU-MFH-2 cells		Original tumor cells
	
	*in vitro*	*in vivo*	
Vimentin	+ + +	+ + +	+ + +
EMA	-	-	-
AE1/AE3	-	-	-
CAM 5.2	-	-	-
Desmin	-	-	-
α-SMA	-	-	-
MSA	-	-	-
S-100 protein	-	-	-
NSE	-	-	-
CD68	+ + +	+ +	+ +
Lysozyme	-	-	+
AAT	-	-	-
ACT	-	-	-
C-Kit	-	-	-

*Abbreviations: *EMA, epithelial membrane antigen; α-SMA, alpha-smooth muscle actin; MSA, muscle-specific actin; NSE, neuron-specific enolase; AAT, alpha-1-antitrypsin; ACT, alpha-1-antichymotrypsin.

+ + +, > 75% positive cells; + +, 15-75% positive cells; +, < 15% positive cells, -, negative reaction.

### Cytogenetic findings

**Figure 4:**
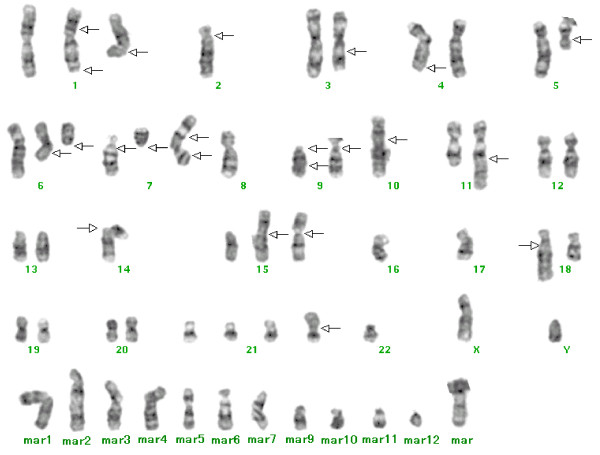
**A representative G-banded karyotype of a metaphase FU-MFH-2 cell, including 12 marker chromosomes**. Arrows indicate the structural chromosome aberrations.

A representative karyotype is shown in Figure 4. FU-MFH-2 displayed a highly complex karyotype with numerous marker chromosomes. The composite karyotype was as follows: 55-61,XY,-X,add(X)(p22.1),add(1)(q11),der(1)add(1)(p13)del(1)(q42),-2,-2,add(2)(p11.1), -3,add(3)(q21),-4,add(4)(q31.1),-5,add(5)(q11.1),del(6)(q11) × 2,del(7)(p11.1), del(7)(q11.1),der(7)add(7)(p22)add(7)(q22),-8,add(9)(p11) × 2, der(9)del(9)(p11)add(9)(q22),-10,add(10)(p13),-11,add(11)(q23),-12,-13,-14,add(14)(p11.1),add(15)(p11.1),add(15)(p11.1),-17,-17,-18,-19,-20,add(20)(q13.1),+add(21)(p11.1),-22,-22, +mar1,+mar2,+mar3,+mar4,+mar5,+mar6,+mar7,+mar8,+mar9,+mar10,+mar11,+mar12 [cp20]. Precisely the same karyotype was recognized in the original tumor cells (data not shown).

### Molecular cytogenetic findings

**Figure 5:**
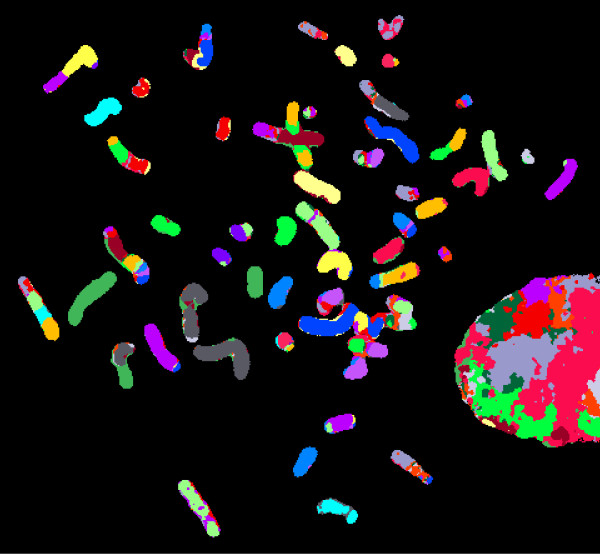
**Multicolor FISH of FU-MFH-2 cell line**. Aberrant chromosomes are displayed in classified color image.

An M-FISH analysis identified 19 structural rearrangements in the FU-MFH-2 cell (Figure 5). Chromosomes 3, 6, 8, 9, 10, and 16 were frequently involved in rearrangements.

**Figure 6:**
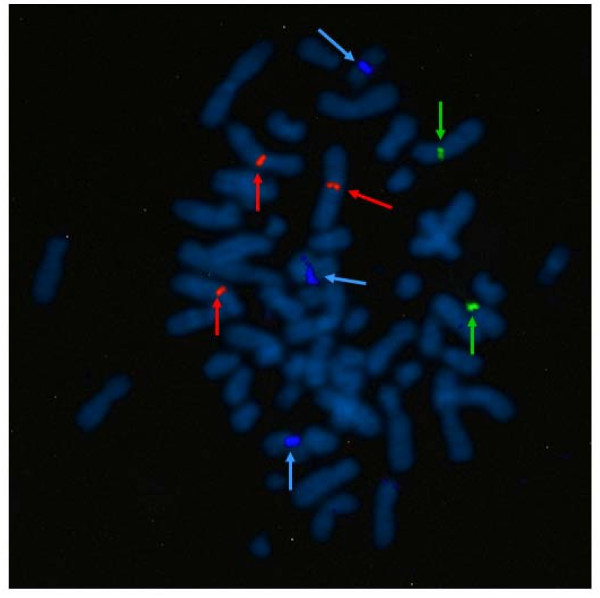
**Multitarget FISH analysis performed on metaphase cells of FU-MFH-2 cell line with the Urovysion™ probe set reveals loss of gold signals indicating homozygous deletions of the 9p21 locus**. Centromeric signals (arrows) of chromosomes 3 (red), 7 (green), and 17 (aqua) are shown.

Urovysion™ FISH revealed homozygous deletions of the 9p21 locus containing the tumor suppressor gene *p16*^*INK4A *^in all analyzed metaphase and interphase cells (Figure 6).

**Figure 7:**
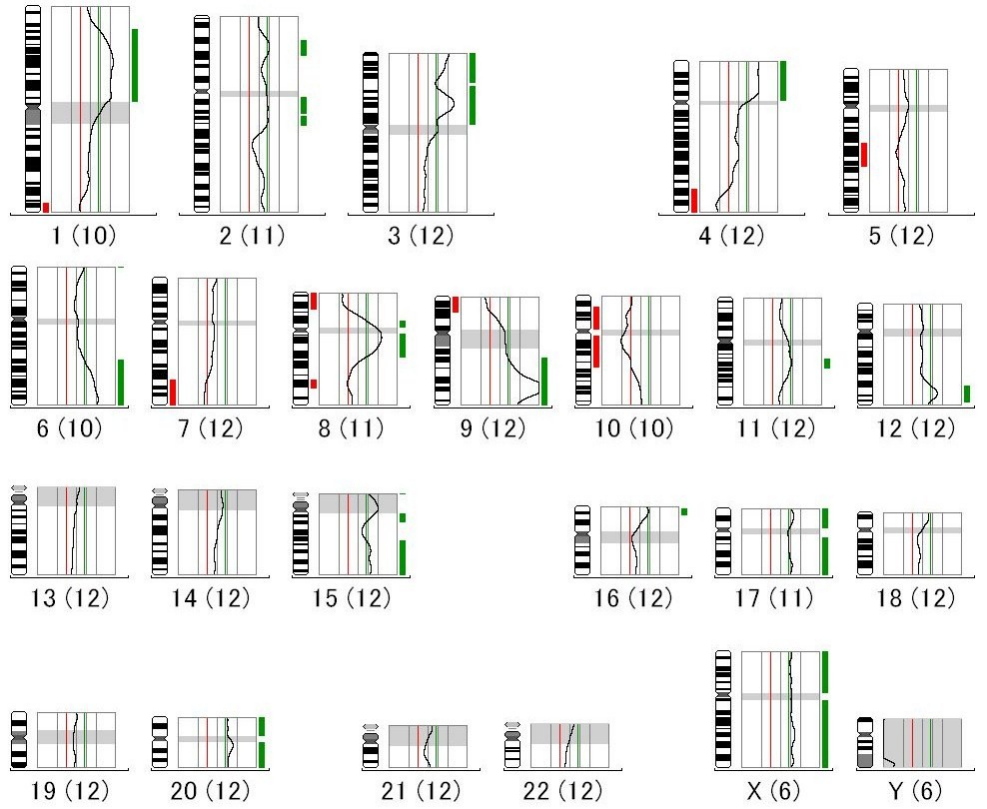
**CGH profile of FU-MFH-2 cell line showing high-level amplification of 9q31-q34, gains of 1p12-p34.3, 2p21, 2q11.2-q21, 3p, 4p, 6q22-qter, 8p11.2, 8q11.2-q21.1, 9q21-qter, 11q13, 12q24, 15q21-qter, 16p13, 17, 20, and X, and losses of 1q43-qter, 4q32-qter, 5q14-q23, 7q32-qter, 8p21-pter, 8q23, 9p21-pter, 10p11.2-p13, and 10q11.2-q22**. The line in the middle (gray) is the baseline ratio (1.0); the left (red) and right (green) lines indicate ratio values of 0.8 and 1.2, respectively. Bars to the left (red) and right (green) of each frame indicate losses and gains, respectively. The terminology 1(10) represents 10 aberrations detected on chromosome 1. The same applies to other chromosomes shown in the profile.

CGH analysis showed similar profiles in the original tumor and FU-MFH-2 cell line. A high-level amplification of 9q31-q34 was observed. Significant gains of DNA sequences were detected in the 1p12-p34.3, 2p21, 2q11.2-q21, 3p, 4p, 6q22-qter, 8p11.2, 8q11.2-q21.1, 9q21-qter, 11q13, 12q24, 15q21-qter, 16p13, 17, 20, and X regions. Significant losses of DNA sequences were detected in the 1q43-qter, 4q32-qter, 5q14-q23, 7q32-qter, 8p21-pter, 8q23, 9p21-pter, 10p11.2-p13, and 10q11.2-q22 regions. This CGH profile is represented in Figure 7.

## Discussion

We established the FU-MFH-2 cell line derived from human pleomorphic MFH and used various analytical methods to characterize this cell line. FU-MFH-2 cells exhibited a spindle and polygonal shape, similar to other pleomorphic MFH cell lines established previously [5,13,15]. The immunophenotype of FU-MFH-2 cells *in vitro *and *in vivo *was similar to that of the original tumor cells. In addition, FU-MFH-2 cells could grow *in vivo *to produce tumors with histopathologic features similar to those of the original tumor in SCID mice. Furthermore, FU-MFH-2 and the original tumor had the same DNA sequence copy number changes by CGH. These findings suggested that this cell line has retained the characteristics of the original tumor.

Cytogenetic analyses of pleomorphic MFH have revealed highly complex karyotypes lacking specific structural or numerical aberrations [1,22]. Recurrent breakpoints are seen in chromosome bands 1p36, 1q11, 1q21, 3p12, 11p11, 17p11, and 19p13 [23-25]. As expected, the FU-MFH-2 cells had complex karyotypes with a number of numerical and structural alterations, including marker chromosomes. Using M-FISH analysis, we were able to decipher the origin of marker chromosomes and complex chromosomal rearrangements. These results emphasize the usefulness of M-FISH in the description of complex changes occurring in pleomorphic MFH cell lines.

CGH studies have indicated that chromosomal gains seem to be more frequent than losses in pleomorphic MFH. Genomic imbalances frequently include gains of 1p31, 5p, 6q22-q24, 7q32, 9q31-q34, 12q13-q15, and 17q and losses of 9p21-pter and 13q14-q21 [26-30]. The FU-MFH-2 cells also had gains of 1p12-p34.3, 6q22-qter, 9q21-qter, and 17 and loss of 9p21-pter. Moreover, a high-level amplification at 9q31-q34 was detected in FU-MFH-2 cells, suggesting a critical role in pleomorphic MFH progression. Interestingly, Tarkkanen et al. reported that gain of 9q32-qter was one of the most frequent genomic imbalances in MFH of bone [31]. Several candidate genes have been mapped to this chromosomal region, including *VAV2*, *ABL1*, *Notch1*, and *Tenascin-C (TNC)*. *VAV2 *is the second member of the VAV guanine nucleotide exchange factor family of oncogenes and is frequently gained in uterine leiomyosarcoma [32]. Translocation-mediated transcriptional activation of tyrosine kinase gene *ABL1 *is implicated in the pathogenesis of chronic myeloid leukemia. *Notch1 *encodes a member of the Notch family and is a transmembrane receptor including an extracellular domain consisting of multiple epidermal growth factor-like repeats and an intracellular domain consisting of multiple, different domain types. The Notch signaling pathway is involved in a variety of cellular differentiation, proliferation, and apoptosis [33]. Enjin et al. reported that human osteosarcoma cell lines and primary human osteosarcoma tumor samples showed significant upregulation of *Notch1 *[34]. *TNC *is an oligomeric glycoprotein of the extracellular matrix that is involved in embryogenesis, tumorigenesis, and angiogenesis. Of note, Franchi et al. reported that TNC expression was found in MFH [35]. However, the role of these genes in the development and progression of pleomorphic MFH is unknown.

The *p16*^*INK4A *^gene is located at 9p21. This gene is frequently mutated or deleted in a variety of tumors and is known to be an important tumor suppressor gene [36]. Frequent deletions of *p16*^*INK4A *^have also been reported in pleomorphic MFH [37]. However, the association between *p16*^*INK4A *^alterations and prognosis in pleomorphic MFH patients remains controversial [1]. In the present study, we decided to examine this gene using metaphase FISH analysis because loss of 9p21-pter was detected by CGH. As expected, homozygous deletion of *p16*^*INK4A *^was observed in FU-MFH-2 cell line. Taken together, these findings suggest that inactivation of *p16*^*INK4A *^by homozygous deletion may be important for pleomorphic MFH development, although not tumor-type specific.

## Conclusion

We described the establishment and characterization of a new permanent human cell line, FU-MFH-2, derived from a metastatic pleomorphic MFH. The FU-MFH-2 will be useful for various biologic and molecular pathogenetic studies of human pleomorphic MFH.

## Competing interests

The authors declare that they have no competing interests.

## Authors' contributions

JN conceived the study and drafted the manuscript. JN and MI carried out the experimental work. TI managed the patient. HI, KN, TI, and MN participated in the design of the study and evaluated the manuscript. All authors read and approved the final manuscript.
